# Fever of Unknown Origin With Normal Inflammatory Markers Due to Latent Pulmonary Embolism and Deep Vein Thrombosis: A Case Report

**DOI:** 10.7759/cureus.42850

**Published:** 2023-08-02

**Authors:** Hisashi Takahashi, Misaki Hanya, Hidetaka Ishino

**Affiliations:** 1 Neurology, North Medical Center, Kyoto Prefectural University of Medicine, Yosano-Cho, JPN; 2 General Internal Medicine, North Medical Center, Kyoto Prefectural University of Medicine, Yosano-Cho, JPN

**Keywords:** c-reactive protein, inflammation, deep venous thrombosis, pulmonary embolism, intermittent fever

## Abstract

An 86-year-old woman who was hospitalized due to cerebral hemorrhage developed an intermittent fever of up to 39.3°C. A computed tomography angiography of the chest with venous runoff to the legs showed pulmonary embolism (PE) and deep vein thrombosis (DVT) of the legs. Intravenous heparin rapidly reduced the fever, indicating that these thrombi were the primary cause of her fever. During her course, white blood cell count and serum C-reactive protein levels were always within normal limits. This case suggested that latent PE and DVT can be a cause of intermittent fever with normal inflammatory markers.

## Introduction

Fever can develop in any patient, but the identification of its primary cause is often challenging. When encountering a fever of unknown origin (FUO), clinicians should consider multiple differential diagnoses and investigate the origins through detailed medical interviews and examinations. In general, infectious diseases, immune-mediated diseases, and cancers induce fever via local or systemic inflammatory responses. Therefore, fever in patients with these diseases is usually accompanied by an elevation of blood inflammatory markers, including the white blood cell (WBC) count, C-reactive protein (CRP), and erythrocyte sedimentation rate (ESR). On the other hand, patients with some diseases can develop fever without elevation of inflammatory markers. Representative examples include patients with non-bacterial meningitis, systemic lupus erythematosus, and heat stroke who develop a fever with normal CRP [1-3]. The discrepancy between fever and normal inflammatory markers can be helpful in detecting the origin of the fever.

Here, we present a case of FUO with normal WBC count and CRP levels, which was suggested to be caused by latent pulmonary embolism (PE) and deep vein thrombosis (DVT).

## Case presentation

An 86-year-old woman was admitted to our hospital due to a cerebral hemorrhage involving the left parieto-occipital lobe. She had hypertension and type 2 diabetes mellitus (DM). Before developing the stroke, she lived alone, and her activities of daily living were fully independent. On admission, she showed mixed aphasia and right hemispatial neglect. She was treated with intravenous nicardipine, tranexamic acid 2 g per day, and carbazochrome sodium sulfonate hydrate 200 mg per day for seven days. Although her neurological symptoms improved gradually, her hospitalization was prolonged. She tended to stay in bed almost all day, and she developed delirium that was treated with risperidone 3 mg per day. She was also taking linagliptin 5 mg per day.

**Figure 1:**
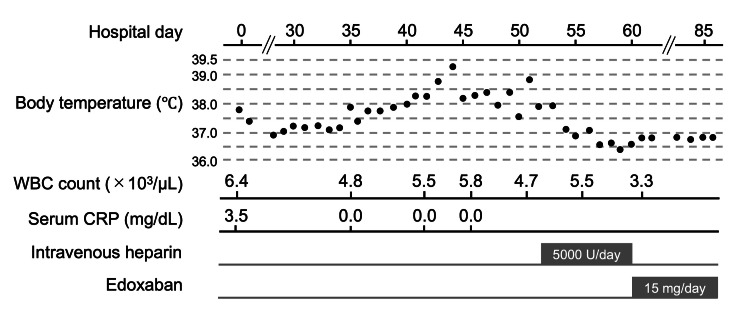
Clinical course of the patient. Black dots represent the maximum body temperature on each day. CRP: C-reactive protein; WBC: white blood cell

On the 35th hospital day, she developed a fever with a temperature of 37.9°C, which normalized during the day. However, intermittent fever subsequently developed and persisted (Figure. 1).

The temperature showed diurnal fluctuation and was mostly up to 38.5°C, though it reached 39.3°C on the 44th hospital day. Acetaminophen and loxoprofen were used on a limited basis when her temperature surpassed 38.0°C, although they seemed to be ineffective. Drugs were suspected to be a cause of the fever. Although risperidone was stopped, her fever continued. On the other hand, her subjective symptoms were relatively mild, and she did not complain of dyspnea, chest pain, or leg swelling. During the course, her blood pressure (<140 mmHg) and heart rate (<100 beats per minute) were normal, whereas her respiratory rate was relatively increased (20-28 breaths per minute), and SpO_2_ on room air fluctuated in the range of 91% to 97%. There were no notable findings on physical examination. Neck stiffness was absent, lymph nodes were not swollen, chest and bowel sounds were normal, no leg edema was observed, and there were no skin rashes.

Blood tests were repeatedly performed during the febrile episodes, and they always showed normal WBC (4.7-5.8 × 10^3^/μL). Serum CRP was also negative each time it was measured (0.0 mg/dL). The results of blood tests are presented in Table 1.

**Table 1:** Blood test findings.

Laboratory investigations	Results	Reference ranges
White blood cell count	4.8 × 10^3^/μL	3.8–8.6 × 10^3^/μL
Neutrophil	74.0%	40.0–77.0%
Hemoglobin	12.6 g/dL	10.0–16.0 g/dL
Hematocrit	38.1%	33–57%
Platelets	212 × 10^3^/μL	140–360 × 10^3^/μL
Sodium	143 mEq/L	135–147 mEq/L
Potassium	3.6 mEq/L	3.3–4.8 mEq/L
Blood urea nitrogen	22.0 mg/dL	8.0–22.0 mg/dL
Serum creatinine	0.43 mg/dL	0.40–0.80 mg/dL
Total bilirubin	0.8 mg/dL	0.2–1.2 mg/dL
Total protein	6.3 g/dL	6.7–8.3 g/dL
Aspartate aminotransferase	18 IU/L	8–38 U/L
Alanine aminotransferase	11 IU/L	4–44 U/L
C-reactive protein	0.0 mg/dL	0.0–0.3 mg/dL
Glucose	173 mg/dL	70–110 mg/dL
Hemoglobin A1c	7.3%	4.6–6.2%
Thyroid-stimulating hormone	2.930 μIU/mL	0.500–5.000 μIU/mL
Free triiodothyronine	2.23 pg/mL	2.30–4.00 pg/mL
Free thyroxine	1.16 ng/dL	0.93–1.70 ng/dL
Prothrombin time	11.7 seconds	10.8–12.8 seconds
Activated partial thromboplastin time	26.9 seconds	14.0–30.0 seconds
D-dimer	15.8 μg/mL	0.0–1.0 μg/mL
Cancer antigen 19-9	69.2 IU/mL	0.0–37.0 U/mL
Carcinoembryonic antigen	8.9 IU/mL	0.0–5.0 U/mL
Cancer antigen 125	12.9 IU/mL	0.0–35.0 U/mL
Soluble interleukin 2 receptor	316 IU/mL	157–474 U/mL
Anti-cardiolipin IgG antibody	Negative	
Anti-cardiolipin IgM antibody	Negative	
Lupus anticoaglant	Negative	
Procalcitonin	0.03 ng/mL	0.00–0.10 ng/mL
T-SPOT	Negative	

Lab tests including liver function tests and electrolytes were normal. Thyroid hormones were within normal limits. Procalcitonin was also normal, and T-SPOT®︎ was negative. Anti-cardiolipin antibodies and lupus anticoagulant were also negative. On the other hand, fasting blood glucose was high (173 mg/dL), and hemoglobin A1c was also elevated (7.3%). cancer antigen 19-9 (69.2 IU/L) and carcinoembryonic antigen (8.9 IU/L) were elevated. Remarkably, D-dimer was significantly elevated (15.8 μg/mL), higher than that on the first hospital day (3.2 μg/mL). Prothrombin time and activated partial thromboplastin time were within normal limits. The urinalysis was unremarkable, and cerebrospinal fluid (CSF) did not show pleocytosis. No pathogenic microorganisms were found in blood, urine, and CSF. Antigens of influenza and coronavirus disease 2019 (COVID-19) were not detected on nasopharyngeal swab examinations. Transthoracic echocardiography detected no valvular diseases, and abnormal shunt flows and vegetations were not observed. Chest X-ray did not show pneumonia or pleural effusion. Head computed tomography (CT) showed no remaining fresh hematoma.

**Figure 2:**
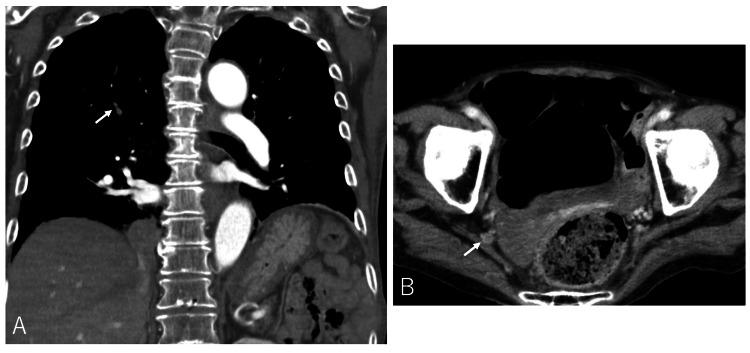
Pulmonary embolism and deep vein thrombosis in the patient. (A) Pulmonary embolism. (B) Deep vein thrombosis. Thrombi are pointed out by white arrows.

From tachypnea and mild hypoxia with normal chest X-ray, and the elevated D-dimer level, thromboembolism was suspected. A CT angiography of the chest with venous runoff to the legs was performed on the 50th hospital day. It showed small PE at pulmonary artery branches and DVT of the right internal iliac vein and bilateral popliteal veins (Figure 2).

At the same time, it showed no aspiration pneumonia, and lung and abdominal tumors were not identified. Continuous intravenous heparin at 5,000 IU per day was started on the 52nd hospital day. Her fever resolved by the 55th hospital day without requiring antibiotics. Considering her old age, low weight (38.5 kg), and recent history of cerebral hemorrhage, oral edoxaban 15 mg per day was started on the 60th hospital day as prophylactic therapy. D-dimer decreased to 2.1 μg/mL on the 63rd hospital day. Thereafter, she was discharged on the 86th hospital day. Oral edoxaban 15 mg per day was continuously prescribed.

## Discussion

We described the case of a hospitalized patient who developed FUO lasting over two weeks. The differential diagnosis included aspiration pneumonia, urinary tract infection, infectious endocarditis, aseptic meningitis, tuberculosis, COVID-19, influenza, drug fever, tumor fever, and fever associated with cerebral hemorrhage; however, these diseases were not detected. A systemic workup eventually identified latent PE and DVT, and anticoagulant therapy with heparin rapidly led to reducing fever, suggesting that they were the primary cause of fever. Although aphasia might affect how patients express themselves, the patient’s subjective symptoms were mild and she did not complain of dyspnea and chest pain, making the diagnosis difficult. On the other hand, her respiratory rate (20-28 breaths per minute) and SpO_2_ (91-95%) exhibited deviations from the expected norm, suggesting the presence of PE. Elevated D-dimer was also helpful in considering thrombosis. Retrospectively, her modified Well’s score was 4.5 (clinical symptom 3, bedridden state 1.5) with elevated D-dimer, recommending contrast-enhanced CT to identify PE [4]. The venous thrombogenesis seemed to be attributed to a decrease in leg exercises and daily activities due to her cerebral hemorrhage and long-term hospitalization. The hemostatic agents such as tranexamic acid and carbazochrome sodium sulfonate hydrate used for her stroke could have also led to blood clotting [5]. Notably, the use of antipsychotic drugs is associated with the development of PE and DVT [6]. In this case, regular risperidone 3 mg per day was used until FUO developed, indicating that this drug might play an essential role in thrombogenesis. This case strongly suggests that hospitalized patients should get exercise regularly and chronic use of antipsychotic drugs should be avoided.

Interestingly, the present patient showed no elevation of inflammatory markers, including the WBC count and CRP, during the febrile episode. The relationships between fever and inflammatory markers in PE and DVT are not fully understood. Previous studies have shown that PE and DVT can induce fever, and they tend to be accompanied by mild elevation of inflammatory markers, including the WBC count, CRP, and ESR [7-9]. As the pathophysiology of the fever, infarction, tissue necrosis, hemorrhage, local vascular inflammation, and atelectasis have been discussed [9]. On the other hand, Murray et al. reported that four of 20 patients with pyrogenic PE did not have elevated WBC count over 10,000/mm^3^, suggesting that PE-related fever can develop without leukocytosis [7]. As for the present patient, the size of the thrombi was relatively small, and the systemic effect of the PE and DVT was not severe, which might result in a weak inflammatory response. Likewise, there are a few case studies describing fever eventually proven to be caused by latent PE and DVT without overlap with other inflammatory disorders (Table 2) [10,11].

**Table 2:** Reported cases of fever due to latent pulmonary embolism and deep vein thrombosis CRP: C-reactive protein; DM: diabetes mellitus; DVT: deep vein thrombosis; ESR: erythrocyte sedimentation rate; HIV: human immunodeficiency virus; HT: hypertension; WBC: white blood cell

Case	Patient	Complications	Diagnosis	Fever	WBC count	CRP	ESR
Borenstein et al. [10]	81-year-old female	Osteoarthritis, fibromyalgia, and rheumatoid arthritis	PE	Intermittent fever up to 39.1°C	11.59 × 10^3^/µL	Mildly elevated	n/a
Saad et al. [11]	A female in her 40s	HIV infection, hypothyroidism, HT, DM	PE, DVT	Persistent fever up to 40°C	6.5–7 × 10^3^/µL	n/a	26 mm/hour
Present case	86-year-old female	Cerebral hemorrhage, HT, DM	PE, DVT	Intermittent fever up to 39.3°C	4.7–5.5 × 10^3^/µL	0.0 mg/dL	n/a

In these patients, the elevation of inflammatory markers was not observed or was limited. The present case and previously reported studies highlight that latent PE and DVT can be a cause of fever with normal inflammatory markers. On the other hand, it should be considered that the present patient might have had some immunological deficits. Older people generally tend to show insufficient immune responses, and she had type 2 DM, which might cause immunosuppression. The use of several antipyretics might have interrupted her inflammatory response. Although several tumor markers were elevated, diagnostic endoscopies and a bone marrow examination were not performed as the patient disagreed. Further case reports and studies are needed to understand the fever and inflammatory markers in patients with PE and DVT.

## Conclusions

The present case suggests that patients with latent PE and DVT can develop FUO with normal WBC count and CRP levels. Clinicians ought to be cognizant of the potentiality of thrombotic complications in patients subjected to prolonged immobility and treated with antipsychotic medications. Fever accompanied by abnormal respiratory rate and SpO_2_, along with elevated D-dimer levels, can provide valuable diagnostic insights concerning thrombosis. This case suggests that PE and DVT belong to a list of diseases that can develop a fever with normal inflammatory markers. Careful attention to these diseases will lead to early diagnosis and treatment and will prevent life-threatening events due to massive PE or paradoxical embolism.
